# Comparative effectiveness of percutaneous epidural adhesiolysis for different sacrum types in patients with chronic pain due to lumbar disc herniation: A propensity score matching analysis

**DOI:** 10.1097/MD.0000000000004647

**Published:** 2016-09-16

**Authors:** Sang Ho Moon, Jun Young Park, Seong-Sik Cho, Hyun-Seok Cho, Jae-Young Lee, Yeon Ju Kim, Seong-Soo Choi

**Affiliations:** a Department of Orthopedic Surgery, Seoul Sacred Heart General Hospital; b Department of Anesthesiology and Pain Medicine, Asan Medical Center, University of Ulsan College of Medicine; c Department of Occupational and Environmental Health, Graduate School of Public Health, Seoul National University, Gwanak-gu, Seoul; d Department of Occupational and Environmental Medicine, Konkuk University Chungju Hospital, Chungju, Republic of Korea.

**Keywords:** chronic low back pain, lumbar intervertebral herniation, percutaneous epidural adhesiolysis, sacral type

## Abstract

For percutaneous epidural adhesiolysis (PEA) in patients with chronic low back and/or leg pain, comparative efficacy of lumbar PEA between the sacral types has not yet been investigated. This study aimed to determine the comparative efficacy of lumbar PEA between the sacral types in chronic pain with lumbosacral herniated intervertebral disc (L-HIVD).

A total of 1158 chronic low back and/or leg pain patients who diagnosed with L-HIVD and underwent PEA between February 2011 and March 2015 were retrospectively examined. All enrolled patients were divided into 2 types: dome-sacral type and flat type. To avoid confounding bias, propensity score analysis was used. Numeric rating scales (NRS) and Patients’ Global Impression of Change (PGIC) were compared between the 2 types at baseline and at 3 months post-PEA.

After conducting a propensity score matching analysis, 114 patients were included in each type. The mean sacral angle significantly differed between the flat-sacral and dome-sacral types (*P* < 0.001). A linear mixed effect model analysis showed that the adjusted NRS score at baseline was 7.58 [95% confidence interval (CI): 7.40–7.76] for the flat-sacral type and 7.47 (95% CI: 7.29–7.64) for the dome-sacral type. The adjusted NRS score after 3 months post-PEA was 4.27 (95% CI: 3.77–4.77) for the flat-sacral type and 3.71 (95% CI: 3.21–4.21) for the dome-sacral type. We detected no significant differences in NRS at baseline (*P* = 0.371) and after 3 months (*P* = 0.121) between the 2 groups. No significant differences were observed in terms of the NRS score between the 2 groups during the 3 months follow-up (omnibus *P* = 0.223). There were no significant differences in PGIC between flat-sacral and dome-sacral types at 3 months after the follow-up period (4.40 ± 2.17 and 4.67 ± 1.88, respectively, *P* = 0.431).

PEA provides sufficient pain relief for chronic pain due to L-HIVD at 3 months postprocedure. The sacral type might not affect the outcome of lumbar PEA in chronic pain associated lumbar HIVD.

## 1 Introduction

Low back and/or radiating leg pain from a lumbosacral herniated intervertebral disc (L-HIVD) is a common medical and social condition.^[1–3]^ The prevalence of persistent low back pain ranges from 35% to 75% at 12 months after an initial onset of an attack.^[2,4]^ In L-HIVD, an intervertebral disc annular is torn and disc material leaks into the epidural space. This process leads to inflammation, which are frequently followed by fibrosis, adhesion, and spinal nerve compression. Consequently, low back pain-induced L-HIVD can occur.^[1–3,5]^ Generally, various conservative therapies are used to treat this pain, including physical therapy, analgesia, and epidural steroid injection.^[6,7]^

Fluoroscopy-guided epidural steroid injection has been used to treat intractable chronic pain caused by L-HIVD.^[8]^ Various epidural injection methods, such as interlaminar, transforaminal, and caudal epidural injections, have been used and studied.^[6,7,9–11]^ However, the benefits of epidural steroid injections for the treatment of chronic low back pain remain controversial.^[6,10–13]^ Because epidural adhesions caused by L-HIVD frequently act as mechanical barriers that prevent medications to reach lesions, the epidural injection method frequently fails to achieve considerable pain relief.^[3,14–17]^

Percutaneous epidural adhesiolysis (PEA) is a minimally invasive therapy in which a catheter is advanced directly into a lesion, such as herniated disc, scar tissue, and the stenotic portion of the spinal canal, which can cause lumbar back pain.^[7,8,12,14,18–24]^ The efficacy of PEA in chronic low back pain has been relatively well investigated. PEA has been used in cases of refractory chronic low back pain^[4,14,25]^ or following postlumbar surgery syndrome (PLSS).^[14,24,26]^ The underlying mechanism of PEA is thought to involve the alleviation of adhesions or compression, which may mechanically hider the direct spread of drugs to the target site.^[6,7,10,14,16,17,23,25–27]^ Therefore, PEA may be effective in pain reduction and functional improvements in patients with chronic low back pain resulting from L-HIVD.^[3,6,7,14,24–27]^ Generally, PEA is performed by a caudal or transforaminal approach. For a caudal approach, an epidural catheter is inserted into the epidural space via the sacral hiatus.^[14,16,25,26]^ However, a caudal approach for catheter insertion is difficult in some patients who have a more concave and angled sacrum. In concave sacral type cases, the procedure was more difficult than in flat-sacral type cases.^[28–32]^ Moreover, from an anatomical perspective, the efficacy of PEA may be low in flat-sacral type than in dome-sacral type cases. To the best of our knowledge, the comparative effectiveness of lumbar PEA between the different sacral types has not been investigated. This study aimed to determine the comparative effects of lumbar PEA between the sacral types in patients with L-HIVD.

## 2 Methods

### 2.1 Patients

This was a single-center, retrospective observational study of an institutional registry that contained the records of 1158 patients who underwent PEA and was conducted with the approval of a Local Ethical Committee of Bundang Cha Hospital (approval number BD 2015-060). Between February 2011 and March 2015, patients with chronic low back pain were examined to ascertain their eligibility.

The patients with chronic low back and/or leg pain lasting more than 3 months due to L-HIVD and the previous failure of treatment such as physiotherapy, exercise, medication, and epidural steroid injection was included for this study. HIVD was confirmed by magnetic resonance imaging (MRI) before PEA. Exclusion criteria for this study included spinal stenosis (congenital or degenerative), compression fracture, and loss to follow-up.

Enrolled patients were divided into 2 groups according to the sacral types. The dome-sacral type was defined as a concave sacral bone that required the placement of pillows under the pelvis rather than the abdomen to expose the sacral hiatus during PEA. Patients with a flat type of sacrum had a pillow placed under their abdomen for the PEA procedure.

### 2.2 Interventions

PEA was performed under fluoroscopic guidance with equipment to monitor blood pressure, electrocardiogram, pulse rate, and pulse oximetry. The patient was placed in a prone position with a pillow according to the sacral type. Fluoroscopy was adjusted over the lumbosacral area so that a caudal approach could be used for both the anteroposterior and lateral views. After sterile preparation and draping of the insertion area, the skin was infiltrated with 1% lidocaine and a 16 G Tuhoy needle was gently advanced under fluoroscopic guidance. Anteroposterior and lateral views were obtained to ensure proper positioning; special care was taken to prevent further possible intravascular or subarachnoid injection. After verifying that there was no aspirated blood or cerebrospinal fluid, a lumbar epidurogram was performed using approximately 5 mL a noniodinated contrast agent (Ultravist, Bayer Korea, Seoul, Korea) to identify the filling defects. After confirmation of adequate radiographic imaging, a pain control manipulator (PCM) catheter (Surgi R&D, Seongnam, Korea) was advanced via the 16 G Tuhoy needle to the site of the filling defect or the known of pathology, as indicated by MRI. Subsequently, adhesiolysis and decompression were carried out by distension with hydrostatic pressure provided by normal saline and mechanical means of the catheter. After adhesiolysis, approximately 3 mL contrast media were injected to confirm satisfactory filling without subarachnoid or intravascular flow. Then, a mixture of 1500 U hyaluronidase and 5 mg dexamethasone in a 6 mL volume of 1% lidocaine was injected via a catheter. Once the procedure ended, the patients were moved to the recovery room. The patient was transferred to a general ward if all vital signs were satisfactory. The initial follow-up was performed 3 months after the procedure. During these periods, all patients received nonsteroidal antiinflammatory drugs and muscle relaxants of equal doses to reduce procedure-related pain.

### 2.3 Outcome measures and follow-up

**Figure 1:**
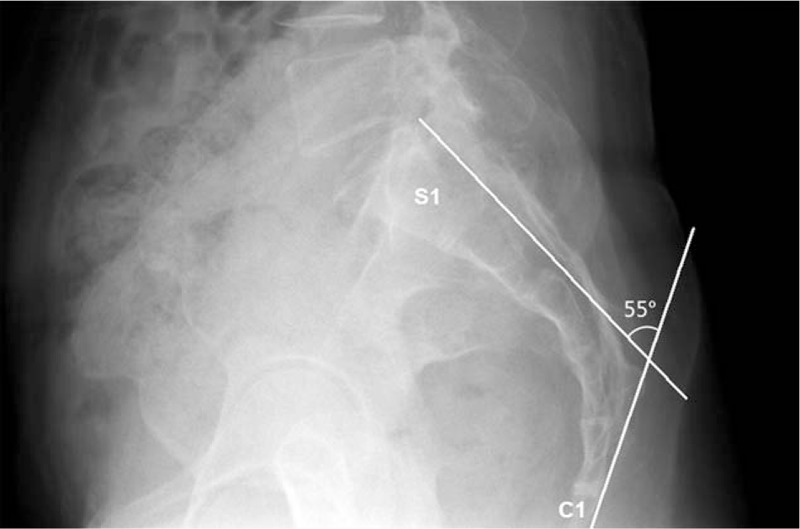
Measurements of the sacral angle using plain radiography. The sacral angle was defined that the angle between the extension line from the posterior body of the first sacral segment (S1) and the extension line from the posterior body of the first coccygeal segment (C1) in a lateral view.

We obtained baseline characteristics, such as age, gender, a history of previous lumbar surgery, a history of previous PEA, and baseline pain intensity. Radiological findings were collected, such as spondylolisthesis, the type of HIVD, the target location, and the number of target levels. Numerical data included age, the number of target levels, and baseline pain intensity. Categorical data included gender, diagnosis, history of a prior operation, PLSS, history of PEA, target level, and target location. The sacral angle was defined as the angle between the extension line from the posterior body of the first sacral segment (S1) and the extension line from the posterior body of the first coccygeal segment (C1) in lateral views (Fig. 1).

Clinical data were collected at baseline and at the 3 months follow-up, consisting of a medical interview and pain assessment. An 11-point numeric rating scale (NRS) was used to assess pain intensity. A 7-point Patients’ Global Impression of Change (PGIC) scale was used to evaluate patient satisfaction and improvement. Adverse events during the procedure and follow-up period were also collected. All patients were asked to provide answers considering the average severity of their symptoms over the previous week.

### 2.4 Statistical analysis

Statistical analyses were performed using SPSS 21.0 for Windows (SPSS, Inc., Chicago, IL) and Stata software version 13.1 (StataCorp LP, College Station, TX). To compare demographic data from the 2 groups, the *χ*^2^ test or Fisher exact test was used to assess categorical data, as appropriate. Student *t*-test or the Mann–Whitney *U* test was used to analyze numerical data, as appropriate. To avoid potential confounding bias, a 1:1 propensity score matching analysis was used to generate a set of matched flat- and dome-sacral types. This was computed for each patient using a logistic regression model that included the following variables: age, gender, type of HIVD, spondylolisthesis, PLSS, history of previous lumbar surgery, history of previous PEA, location of target, number of target levels, and baseline pain intensity. After propensity score matching, the Wilcoxon signed rank test was used to analyze numerical data. Categorical data were analyzed using the McNemar test. As data loss resulting from missing values from electronic databases was expected, the linear mixed effect model (LMEM) was used to compare changes within and between groups in terms of NRS pain scores at baseline and at 3 months post-PEA. PGIC were compared between the 2 types at 3 months post-PEA. A value of *P* < 0.05 was used as a threshold for statistically significant differences.

## 3 Results

**Figure 2:**
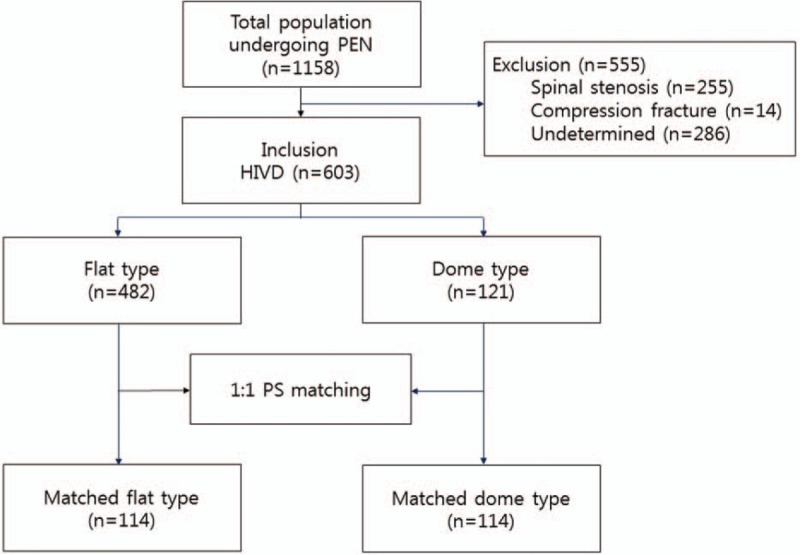
Flow diagram of the study.

As shown in Fig. 2, a total of 1158 patients were admitted for PEA between November 2011 and April 2015. This group included 603 patients who fulfilled our inclusion criteria with radiographic evidence of L-HIVD and were enrolled. Among the 555 excluded patients, 255 was spinal stenosis, 14 had a compression fracture, and 286 was undetermined that lacked a medical record or lost to follow-up. Among the included patients, there were 482 and 121 patients with the flat-sacral and dome-sacral types, respectively.

The baseline characteristics of patients with flat-sacral and dome-sacral types are presented in Table 1. Compared with flat-sacral type patients, patients with the dome-sacral type were older [52.5 (41.0–61.0) years vs 59.0 (49.0–68.0) years, respectively, *P* < 0.001] and more frequently had history of PLSS [33 (6.8%) vs 15 (12.4%), respectively, *P* = 0.044]. Following 1:1 propensity score matching analysis, a total of 114 patients were included for each type. After propensity score matching, no significant differences in demographic data were detected between the 2 groups for all of the variables (Table 2).

**Table 1:**
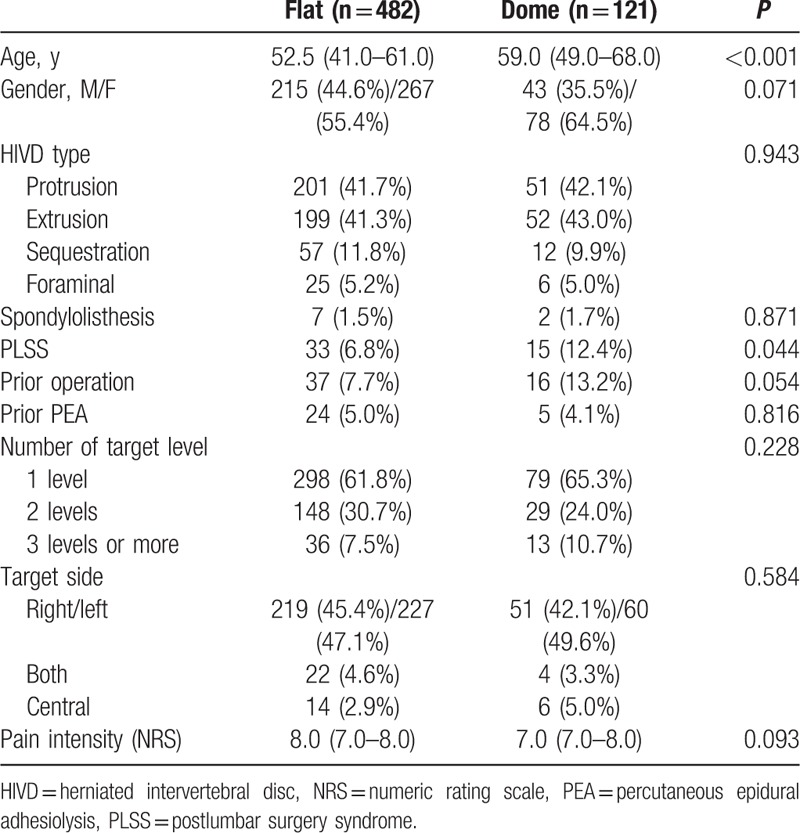
Baseline characteristics of the lumbar disc herniation patients with a flat-sacral or dome-sacral type before a propensity score matching.

**Table 2:**
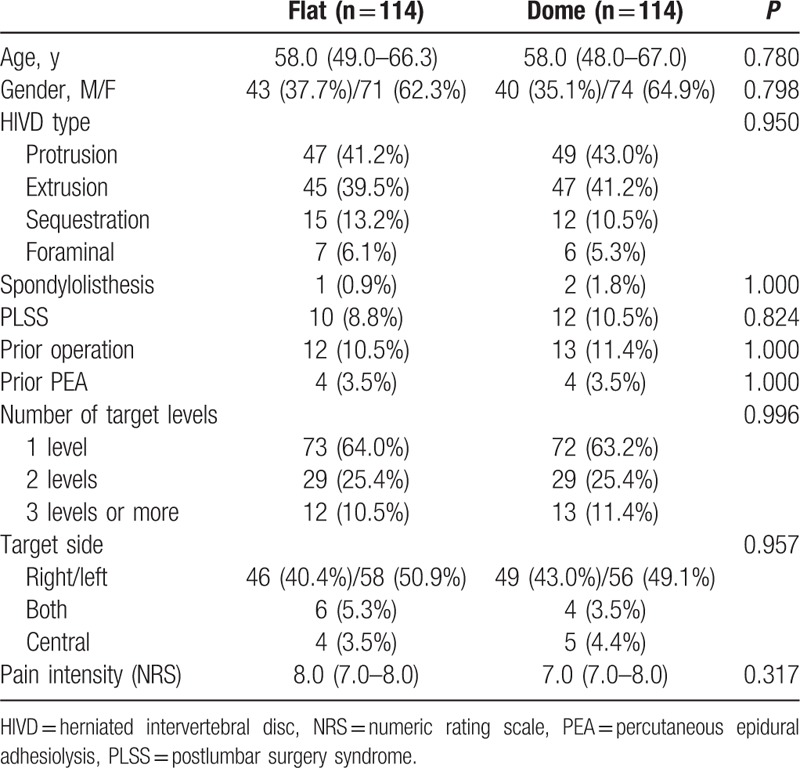
Baseline characteristics of the lumbar disc herniation patients with a flat-sacral or dome-sacral type after a 1:1 propensity score matching.

The median sacral angle between flat-sacral and dome-sacral type after matching was 35.3 (27.5–41.1) and 49.3 (47.8–55.7) degrees, respectively (*P* < 0.001). The difference between the flat and dome types was −18.76 [95% confidence interval (CI): −20.73 to 16.72]. The NRS values at baseline and 3 months after PEA between both types are listed in Table 3. In both the flat-sacral and dome-sacral groups, significant differences were observed in the 3 months after PEA compared with the baseline NRS score (*P* < 0.001, respectively). LMEM analysis indicated that the adjusted prediction of the NRS score at baseline was 7.58 (95% CI: 7.40–7.76) for the flat-sacral type and 7.47 (95% CI: 7.29–7.64) for the dome-sacral type. At 3 months after PEA, the adjusted NRS score was 4.27 (95% CI: 3.77–4.77) for the flat-sacral type and 3.71 (95% CI: 3.21–4.21) for the dome-sacral type. There were no significant differences detected between the 2 groups in NRS at baseline (*P* = 0.371) or after 3 months (*P* = 0.121). No significant differences were observed for the NRS score between the 2 groups during the 3 months follow-up period (omnibus *P* = 0.223).

**Table 3:**
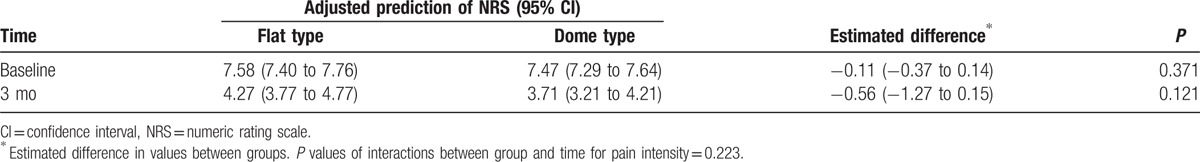
Adjusted predictions of pain intensity and differences after percutaneous epidural adhesiolysis between lumbar disc herniation patients with the flat-sacral and dome-sacral type.

The PGIC values at 3 months after PEA between both types are provided in Table 4. The estimated PGIC at 3 months was 4.40 ± 2.17 for the flat-sacral type and 4.67 ± 1.88 for the dome-sacral type. The estimated difference between the 2 groups in PGIC values at 3 months was −0.28 (95% CI: −0.97 to 0.42); this difference was not statistically significant (*P* = 0.431).

**Table 4:**
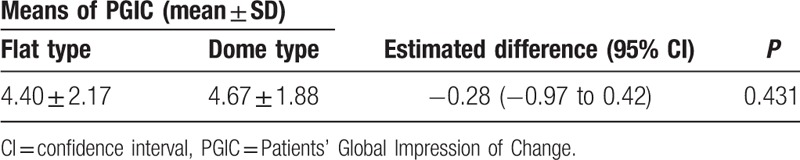
Mean PGIC values and differences after percutaneous epidural adhesiolysis between lumbar disc herniation patients with the flat-sacral and dome-sacral type.

## 4 Discussion

Our present report is the first study to show the comparative effectiveness of PEA between sacrum types in patients with chronic back and/or leg pain associated with L-HIVD. We found that PEA provided sufficient pain relief in patients who had back and/or radiating leg pain resulting from L-HIVD at 3 months after the procedure. We also found no significant differences between the 2 sacral types regarding the NRS score and PGIC during 3 months follow-up period.

Low back pain can be caused by a herniated disc of lumbar spine. Such a disc can become painful as a result of 2 mechanisms. Inflammatory chemicals, such as phospholipases, tumor necrosis factor, or nitric oxide, within the nucleus pulposus as a result of by annular injury, can induce chemical nociception. Additionally, increased stress in the disc can represent a stimulus for mechanical nociception.^[2,5,33]^

PEA has been used to treat chronic pain that is refractory to conventional treatment, and has been known to have better clinical efficacy compared with other treatments, such as epidural steroid injection, because it eliminates adhesions and fibrosis that may prevent the spread of medications resulting from the placement of the catheter tip to the lesion.^[4,10,16,17,22–26]^ In the end, this enables the administration of an adequate volume of steroid or another solution to the appropriate target area.^[4,16,17,24–26]^ If a medication is injected to treat the site of nerve pathology, the therapeutic effects might be maximized.^[20,21]^ Consequently, most physicians attempt to place a catheter at the site of pathology. However, the targeted position cannot always be achieved in clinical practice. In the experience of our group and others, the procedure might be difficult in dome-sacral type patients.^[28–32,34]^ Therefore, it is thought that the effectiveness of PEA may be affected by the sacral angle.

Although no data are available regarding the sacral type in PEA, there are some interesting studies. Evcik and Yücel showed that there was no significant difference or correlation in lumbosacral or sacral horizontal angles and spinal mobility between the acute and chronic lumbar back pain groups.^[30]^ Korovessis et al^[34]^ reported that L5-S1 segmental lordosis was greater in patients with chronic pain. It is known that there is a direct correlation between the lumbosacral angle and lumbar lordosis. Additionally, there is controversy between the vertical angle of sacral curvature (VASC) and L-HIVD.^[30,35]^ Kanat et al^[29]^ found that the VASC is a risk factor in females with both chronic back pain and lumbar disc herniation. However, Ghasemi et al studied that VASC is not an independent risk factor of L-HIVD. In our present study, the sacral angle was defined as the angle between the extension line from the posterior body of the first sacral segment (S1) and the extension line from the posterior body of the first coccygeal segment (C1) in lateral views. Our sacral angle is determined by both lumbar lordosis associated with the sacral horizontal angle and the kyphotic components of the sacral curvature associated with VASC.^[28–30]^ Considering PEA, we assume that both sacral curvature and lumbar lordosis may have anatomical importance for catheter placement and drug delivery.

Notably, in our current analysis, PEA provided sufficient pain relief to patients who experienced low back pain resulting from L-HIVD at 3 months after the procedure, irrespective of the sacral type. It thus appears that the sacral type does not affect the outcome of PEA. This finding may suggest that the sacral angle determined by the kyphotic sacral curvature and lumbar lordosis may not influence to place of the catheter, despite the technical difficulty in conducting this procedure in dome-sacral type patients. Therefore, once the epidural space is ensured via the sacral hiatus, irrespective of the technically difficulty, a successful outcome of PEA may be achieved.

There were several limitations to our present study. First, the duration of the follow-up period was relatively brief, and the power of this study was weakened by the short follow-up period. Second, this was not a controlled, prospective study. This study was a retrospective design. Third, demographic data between the 2 study groups showed significant differences for some variables, such as age and a history of PLSS. Although a propensity score analysis was used to avoid a potential confounding bias, we are not certain that there was no unknown covariate as we did not perform a randomized controlled study. Another limitation resulted from the inclusion criteria. We initially performed the PEA in all patients. However, we enrolled patients with L-HIVD and excluded patients with spinal stenosis because we were concerned that spinal stenosis could independently affect PEA efficacy. Additionally, we excluded many patients who dropped out or were lost to follow-up.

Despite these limitations, our present study suggests that PEA may be a potential treatment option for patients with L-HIVD, even if there is some difficulty in performing this procedure in sacral type patients. Moreover, the sacral type does not reduce effectiveness of PEA in patients with HIVD.

## 5 Conclusions

PEA provides sufficient pain relief for back pain due to L-HIVD at 3 months postprocedure. The sacral type may not affect the NRS score at 3 months after PEA in L-HIVD patients. Additionally, the PGIC score at 3 months post-PEA is not affected by the sacral type.
